# Positive association between physical activity and *PER3* expression in older adults

**DOI:** 10.1038/srep39771

**Published:** 2017-01-03

**Authors:** Masaki Takahashi, Atsushi Haraguchi, Yu Tahara, Natsumi Aoki, Mayuko Fukazawa, Kumpei Tanisawa, Tomoko Ito, Takashi Nakaoka, Mitsuru Higuchi, Shigenobu Shibata

**Affiliations:** 1 Faculty of Science and Engineering, Waseda Univesity, Shinjuku, 162-8480, Japan; 2 Graduate School of Advanced Science and Engineering, Waseda Univesity, Shinjuku, 162-8480, Japan; 3 Waseda Institute for Advanced Study, Waseda University, Shinjuku, 162-8480, Japan; 4 Faculty of Sports Sciences, Waseda University, Tokorozawa, 359-1192, Japan; 5 Department of Medicine, Tokyo Women’s Medical University, Medical Center East, Arakawa, Tokyo, 116-8567, Japan; 6 Institute of Advanced Active Aging Research, Waseda University, Tokorozawa, 359-1192, Japan

## Abstract

The circadian clock regulates many physiological functions including physical activity and feeding patterns. In addition, scheduled exercise and feeding themselves can affect the circadian clock. The purpose of the present study was to investigate the relationship between physical/feeding activity and expression of clock genes in hair follicle cells in older adults. Twenty adult men (age, 68 ± 7 years, mean ± SE) were examined in this cross-sectional study. Prior to hair follicle cell collection, the participants were asked to wear a uniaxial accelerometer for one week. The timings of breakfast, lunch, and dinner were also recorded. Hair follicle cells were then collected over a 24 h period at 4 h intervals. The amplitude of *PER3* expression was positively correlated with moderate and vigorous physical activity (*r* = 0.582, *p* = 0.007) and peak oxygen uptake (*r* = 0.481, *p* = 0.032), but these correlations were not observed for *NR1D1* or *NR1D2*. No association was noted between meal times and the amplitude or the acrophase for any of these three clock genes. These findings suggest that rhythmic expression of the circadian clock gene *PER3* is associated with the amount of daily physical activity and physical fitness in older adults.

The circadian clock system consists of a central clock located in the suprachiasmatic nucleus of the hypothalamus and peripheral clocks located in peripheral tissues, all of which have an approximately 24 h rhythm in mammals^1^. Circadian clock genes regulate the daily rhythms of physiology and behavior such as locomotor activity, feeding time, sleep-wake cycle, body temperature, metabolism, alertness, and cognition in all mammals including humans^1^,^2^,^3^,^4^. Scheduled feeding and exercise in mice can entrain peripheral circadian rhythms under light-dark conditions^5^. In addition, scheduled feeding and exercise in mice can restore to normal the amplitude reduction and arrythmicity of clock gene expression created under constant light conditions^6^. Recently, one study in humans has reported that clock gene expression in subcutaneous fat is regulated by body weight changes and is associated with BMI, serum cholesterol levels and expression of metabolic and inflammatory genes^2^. Another study in humans has shown that heavy physical exercise might influence the circadian phase of clock gene expression in hair follicle cells^7^. Thus, there are strong relationships between physical/feeding activities and clock gene expression in the peripheral organs of mice and human.

In aging, it has been reported that body temperature amplitude is progressively blunted in humans^8^. In mice, aging also causes a functional deterioration in circadian systems including the expression of circadian clock genes^9^,^10^,^11^. In fact, a relationship between abnormal circadian clock functions and age-related cognitive deficits and Alzheimer’s disease has been demonstrated^12^,^13^,^14^. One study reported that the circadian rhythmicity of core body temperature is correlated with a higher level of physical activity in older adults^15^. However, little is known about the relationship between daily physical activity and/or aerobic capacity and the rhythmic expression of circadian clock genes in older adults. From the viewpoint of abnormal circadian rhythm and prevention of various diseases with aging, it may be important to elevate the amplitude of clock gene expression by increasing physical activity.

In order to evaluate clock gene expression in humans, several different sampling methods have been used including measurement of blood metabolites and, collection of white blood cells or oral mucosa cells^16^,^17^,^18^. To solve the problem of obtaining peripheral tissues in a non-invasive way, several studies have reported the use of hair follicle cells as a quick and convenient means for evaluating human circadian clock genes^19^,^20^. Moreover, Akashi *et al*. have reported that circadian fluctuations in *PER3, NR1D1*, and *NR1D2* in hair follicle cells could be used to evaluate circadian rhythms in humans^19^,^21^. *NR1D1* and *NR1D2* (also known as *Rev-erb* alpha and beta) are nuclear receptors that regulate a number of physiological functions including circadian rhythm, lipid metabolism, immune function, and cellular differentiation^22^,^23^,^24^. Although it is difficult to clarify the physiological function of PER3, some studies have indicated that a *PER3* variant is correlated with the circadian phenotype (Morningness-Eveningness score; MEQ), sleep phase disorder, and sleep homeostasis^25^,^26^,^27^. Recently, one study has reported that PER3 variant causes a circadian phenotype, and is associated with a seasonal mood trait^28^. The purpose of the present study was to investigate the relationship between physical/feeding activity and expression of clock genes in hair follicle cells in older adults. We hypothesized that an increase in daily physical activity and time of breakfast would both be correlated with clock gene expression patterns.

## Results

### The rhythm of clock gene expression in hair follicle cells

**Figure 1:**
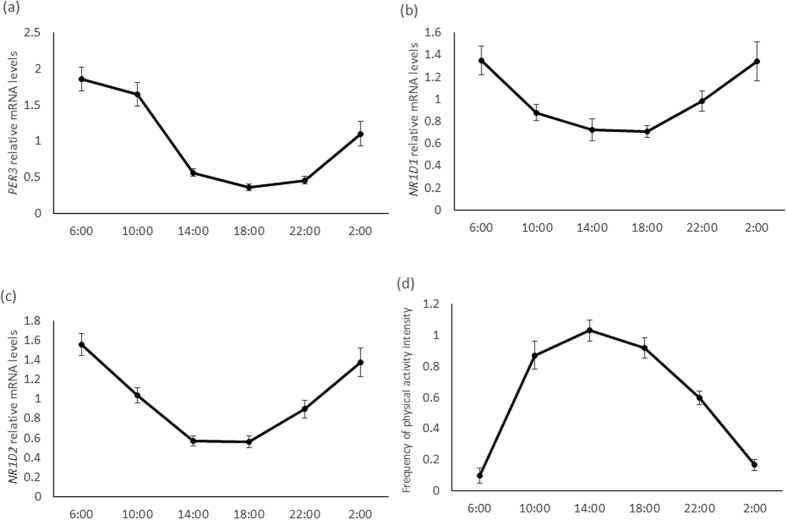
The diurnal *PER3* (**a**), *NR1D1* (**b**), and *NR1D2* (**c**) expression in hair follicle cells and pattern of physical activity (d) (n = 20). Data are means ± SE. Main effect of time (*PER3*, P = 0.001; *NR1D1*, P = 0.001, *NR1D2*, P = 0.001; physical activity, P = 0.001; one-way ANOVA).

The expression rhythms of three clock genes, namely *PER3, NR1D1*, and *NR1D2,* as well as the house keeping gene 18s-*rRNA,* were investigated in this study. One-way ANOVA revealed a significant main effect of time for *PER3, NR1D1,* and *NR1D2* expression (p = 0.001 for each) (Fig. 1a,b,c). However, there are no significant main effect of time for 18s-rRNA (Figure S1). The results of cosinor analysis, including the amplitude and the acrophase values, are presented in Table 1. The individual amplitude and acrophase data are shown in Table S1.

### Relationship between physical activity and the amplitude or the acrophase of clock gene expression rhythm

The amount of moderate to vigorous physical activity (MVPA) and step counts detected by the accelerometer were 170 ± 26 min/week (mean ± SE, range: 23–423 min/week) and 8389 ± 802 step/count (mean ± SE, range: 2847–15782 step/day) respectively. The 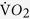 peak was 25 ± 1 ml/min (mean ± SE, range: 16–36 ml/min).

**Figure 2:**
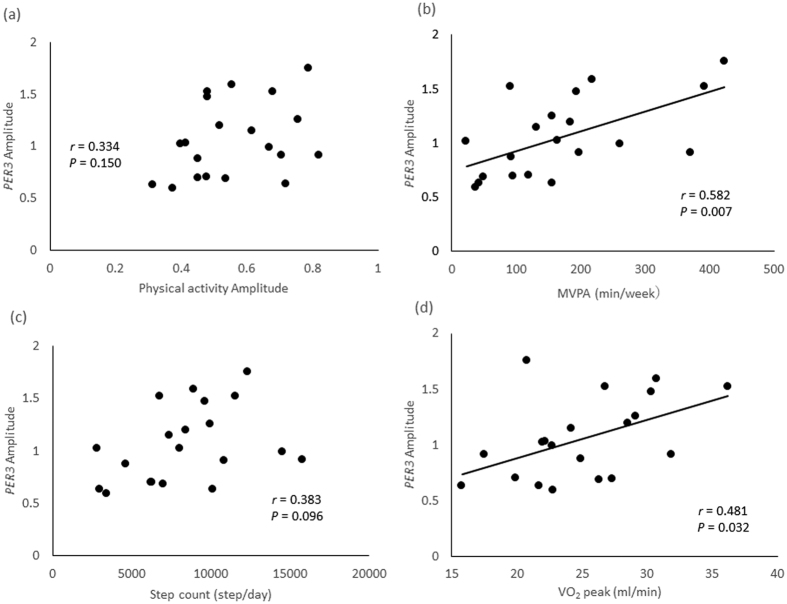
The relationship across all participants between amplitude of physical activity (**a**), moderate to vigorous physical activity (MVPA) levels (**b**), step count (**c**), 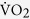 peak (**d**) and expression of *PER3* (n = 20). The correlation coefficient ((**a**), *P* = 0.150, (b), *P* = 0.007, (**c**), *P* = 0.096, (**d**), *P* = 0.032; Pearson’s correlation coefficient).

Patterns of physical activity were also investigated in this study. One-way ANOVA revealed a significant main effect of time for patterns of physical activity (Fig. 1d). The patterns of physical activity were not associated with the amplitude of clock gene expression (*PER3, NR1D1*, and *NR1D2*) (Fig. 2a) (Figure S2a,b).

The amplitude of *PER3* expression was positively correlated with MVPA (*r* = 0.582, *p* = 0.007) (Fig. 2b, Table S2) and tended to be positively correlated with step counts (*r* = 0.383, *p* = 0.096) (Fig. 2c). The acrophase of *PER3* expression was positively associated with MVPA, but not with step counts (Table S3). However, MVPA and step counts were not associated with either the amplitude or the acrophase of *NR1D1* and *NR1D2* expression (Tables S2 and S3).

The amplitude of *PER3* expression was found to be higher in the active group compared to the inactive group (p = 0.039) (Figure S2a), but this was not the case for the amplitude of *NR1D1* and *NR1D2* expression (Figure S2b,c).

### Relationship between 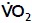 peak and the amplitude or the acrophase of clock gene expression

The amplitude but not the acrophase of *PER3* expression was positively correlated with 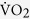 peak (*r* = 0.481, *p* = 0.032) (Fig. 2d, Tables S2 and S3). In contrast, the 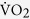 peak was not associated with either the amplitude or the acrophase of *NR1D1* or *NR1D2* expression (Tables S2 and S3).

### Timing of diet and MEQ score and its effect on the amplitude or the acrophase of clock gene expression

The effect of meal times was also evaluated in this study. Breakfast time (range; 5:30–10:00), lunch time (range; 11:30–13:30), and dinner time (range; 17:00–20:00) were not correlated with the amplitude or acrophase of *PER3, NR1D1*, or *NR1D2* expression (Tables S2 and S3). MEQ scores (age-adjusted MEQ score range; 38–60) were not correlated with the amplitude or acrophase of *PER3, NR1D1*, or *NR1D2* expression (Tables S2 and S3).

## Discussion

To the best of our knowledge, the present study is the first to examine the relationship between the amount of daily physical activity/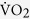 peak and the expression of clock genes in humans. The main findings are that, in older adults, there was a positive correlation between MVPA and the amplitude/acrophase of *PER3*, and a positive correlation between 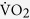 peak and *PER3* amplitude. These findings indicate that elevating the aerobic capacity by increasing daily physical activity may affect the internal circadian clock system driven by *PER3*.

It is well documented that circadian rhythms regulate physiological and biological processes involved in the sleep-wake cycle, body temperature, metabolism, alertness, and cognition^1^,^2^,^3^,^4^. Akashi *et al*. have reported that assessing peripheral clock gene expression in hair follicle cells could be an effective tool for evaluating human circadian rhythms^19^,^21^. These studies showed that the peak times of expression of *PER3, NR1D1*, and *NR1D2* were correlated with the average wake time. Moreover, Watanabe *et al*. have demonstrated that the phase of *PER3* expression in leukocytes significantly with that seen in beard hair follicle cells^20^. In the present study, the expression patterns of *PER3, NR1D1*, and *NR1D2* observed are consistent with those reported in previous studies^19^,^20^. Our findings convincingly demonstrate that examining clock gene expression using hair follicle cells is a convenient method in old adults.

Several studies have reported that the circadian system can be influenced by exercise and physical activity^29^,^30^,^31^. One study has shown that circadian clock gene expression was phase-delayed following high-intensity exercise training in the evening^7^. In addition, amplitudes in body temperature are higher in active adults than in sedentary adults^31^,^32^. Recently, it has been shown that a high level of aerobic capacity may be related to the elevation of rhythmicity in body temperature in older adults^15^. However, few studies are available addressing the relationship between daily physical activity status/physical fitness and circadian expression of clock genes in older adults. The amplitude of circadian clock gene expression is thought to indicate the strength of the circadian rhythm, which decreases with aging^8^. In the present study, the amplitude of *PER3* expression was positively correlated with the amount of moderate and vigorous intensity physical activity in older adults. Interestingly, *PER3* expression was higher in active older adults who adhered to physical activity guidelines for health (i.e. >150 min/week) than in inactive adults.

Although the mechanism is unclear at present, the circadian clocks in peripheral tissues might be induced by exercise or physical activity^33^,^34^. A previous study has reported that scheduled exercise in mice altered the daily rhythm of clock gene expression^33^. Increasing physical activity by scheduled exercise elevated the amplitude and phase of both heart rate and body temperature rhythms. Additionally, the amplitude of PER2:LUCIFERASE expression rhythms in the SCN and peripheral tissues of Per2:Luc knock-in mice were increased by exercise. Consistent with the previous study in mice, the present study found that the amplitude of *PER3* expression was positively correlated with the amount of physical activity in older adults.

The amplitude of *PER3* expression was also positively correlated with the 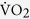 peak in older adults. It is worth noting that the mean values observed in the present study are consistent with data previously observed in sedentary older adults^35^. The 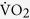 peak expresses the aerobic capacity, which declines with aging^36^. A previous study has reported that 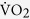
max, which is one of the aerobic capacity parameters, declines by approximately 10% per decade^36^. Moreover, especially in older adults, overall physical fitness and aerobic capacity is dependent on daily physical activity. In fact, the 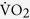 peak in the present study was positively correlated with the amount of moderate and vigorous physical activity. Thus, it may be important to elevate aerobic fitness and capacity or increasing daily physical activity for elevating circadian rhythm amplitude.

We also assessed the pattern of physical activity and its relation to circadian clock gene expression. Although the peak time of physical activity was 3:00 PM in all participants, there was no association between the peak time of daily physical activity and the acrophase of *PER3, NR1D1, and NR1D2* expression. Therefore, regular physical activity may increase the amplitude of clock genes without influencing the time of physical activity. On the other hand, the acrophase of *PER3* expression was positively association with MVPA. In mice, high intensity treadmill exercise could entrain peripheral clocks^5^. MVPA may therefore be sufficient physical activity for entrainment of human peripheral clocks. In the present study, there was no association between amplitude/acrophase of clock gene expression and the timing of breakfast, lunch, and dinner. Although feeding pattern is well known to be associated with clock phase in murine peripheral clocks^37^, feeding time appears not to be an important factor in setting the amplitude/acrophase in human peripheral clocks.

Watanabe *et al*. have reported that the circadian rhythmicity of *PER3* in hair follicle cells assessed by the cosinor method program was not observed in half of the subjects^20^. On the other hand, Akashi *et al*., again using the cosinor method program, have shown that the peak time of *PER3, NR1D1*, and *NR1D2* expression in hair follicle cells correlated with the average wakefulness time^19^. Therefore, the acrophase calculated using the cosinor method program may still be considered valid.

The present study has, however, some limitations. Firstly, it is difficult to separate cause and effect, because this study was a cross-sectional study. Thus, an intervention study would be required to examine the effect of increased physical activity on clock gene expression. Secondly, it is unclear if there is an effect of other external synchronizers such as light on clock gene expression in this study. Although active participants may spend more time outside, and therefore get more exposure to sunlight, further investigation will be needed to take light exposure into consideration, by using a device that could accurately measure light exposure. Thirdly, it is unclear whether the effects of physical activity on other clock genes such as PER2 and BMAL-1 and clock genes in other tissues is similar or different. Additional research will be required to examine the effects of physical activity on circadian clock genes obtained from the blood samples, or oral mucosa cells compared to hair follicle cells. Regarding the expression of PER2 and BMAL-1 in hair follicle cells, a previous study has shown that the expression of PER2 oscillated with lower amplitude, and only slight oscillations were detected for BMAL-1^19^. Thus, monitoring these clock genes in hair follicle cells may be less useful in evaluating circadian rhythms in human. Finally, it has been difficult to clarify the physiological and biological function of *PER3*. In fact, understanding the role of *PER3* in humans has been a focus for the last several years. In this regard, several studies have indicated that polymorphisms in the *PER3* gene in humans may be related to the pathogenesis of delayed sleep phase syndrome^26^,^38^. Recently, one genome-wide association analysis with 89,283 subjects reported that *PER3* variants are associated with self-reporting of being a ‘morning person’^39^. Another study has also demonstrated that a *PER3* variant causes a circadian phenotype and was associated with a seasonal mood trait^28^. Taken together these reports indicate that *PER3* may have an important role in the development of chronotype, sleep phenotype, and depression and this will likely guide future research.

In conclusion, our study has revealed an association between *PER3* expression and daily physical activity in a sample of older adults. We have also demonstrated higher levels of *PER3* in older men who have higher aerobic fitness.

## Methods

### Participants

Twenty-eight participants were recruited from the general population from two local communities in the Tokorozawa metropolitan area. All participants in this study were men. The participants in the present study did not perform any shift-work or travel for one week prior to hair follicle cell collection. None of the participants were involved in athletics although some were recreationally active. Eight participants were subsequently excluded from the data analysis. The exclusion criteria included yielding an insufficient total amount of RNA (<40 ng/μL), and having insufficient accelerometer wear time (see “Physical activity measurement” section below). The physical characteristics of the participants are shown in Table 2. This study was conducted according to the guidelines laid down in the Declaration of Helsinki and was approved by the ethics committees of Waseda University (2014-G003). Informed consent was obtained from all participants following a detailed description of the experiment.

### Baseline measurements of anthropometry, diet time, sleep profile, chronotype

For all participants, anthropometric variables were measured before physical activity measurements were performed Body mass was measured to the nearest 0.1 kg using a digital scale (Karada Scan HBF-701, Omron Co., Japan). Height was measured to the nearest 0.1 cm using a wall-mounted stadiometer (YL-65, Yagami, Co., Japan). Body mass index (BMI) was calculated as weight in kilograms divided by the square of height in meters. Waist circumference was measured to the nearest 0.1 cm at the level of the umbilicus using a flexible plastic tape.

Participants were asked to respond to questions relating to diet and sleep such as “What time do you usually eat breakfast, lunch, and dinner?”, “During the last month, what time do you usually go to sleep and wake up?”, “How many hours do you usually sleep per day during the last month?”.

To assess chronotype, we used the Japanese version of the Horne-Ostberg Morningness-Eveningness Questionnaire (MEQ)^40^,^41^. This tool has been validated in a Japanese population^41^,^42^. The MEQ consisted of 19 questions related to preferred sleep time and daily performance (e.g. What would be the best time to perform hard physical work?). Because diurnal preference changes with age, MEQ scores were adjusted by age (age-adjusted MEQ score, MEQ score + 0.3512 × (39.212–age))^42^. The scores ranged from 16 to 86. Based on age-adjusted MEQ scores, participants were classified into three chronotype groups; either morningness (score 59–86), intermediate (score 42–58), or eveningness (score 16–41). The participants in the present study were classified as follows: 1 morningness, 19 intermediate.

### Baseline measurements and determination of V.O_2_ 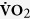 peak

At the first laboratory visit, the subjects’ height, weight, and percent body fat were measured by the same experimenter. After these measurements were taken, peak oxygen uptake (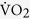 peak) was measured using a maximal graded exercise test on a cycle ergometer (Ergomedic 828E-Monark, Varberg, Sweden). The graded cycle exercise began at a workload of 30 W, which was increased by 15 W every minute until the subject could no longer maintain the required pedaling frequency of 60 rpm. During exercise, a 12-lead electrocardiogram was electronically recorded (Stress Test System ML-6500; Fukuda Denshi, Japan), and heart rate was derived from the R-R interval. In addition, ratings of perceived exertion (RPE) were determined at the end of each minute of exercise Pulmonary gas exchange (oxygen uptake [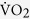], carbon dioxide output [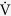
CO_2_], minute ventilation [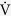
E], and respiratory exchange ratio [RER]) were determined breath-by-breath using a gas analyzer (AE-300S; Minato Medical Science, Japan).

To ascertain that 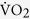 peak was attained, at least two of the following four criteria had to be met: (1) 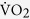 plateaued despite increasing exercise intensity, (2) the highest HR measured during the last minute of exercise was >90% of the predicted maximal heart rate (using the formula 220 − age [in years]), (3) the highest RER was >1.10 during the final stage of incremental exercise, and (4) the subject achieved an RPE of ≥18.

### Physical activity measurement

In order to determine daily physical activity levels, participants were asked to wear a uniaxial accelerometer (Lifecorder-EX; Suzuken Co. Ltd., Nagoya, Japan) for one week prior to hair follicle cell collection. A number of studies have reported that the Lifecorder-EX is considered as a validated accelerometer to evaluate and monitor step counts and physical activity levels^43^,^44^. The device reports the intensity of the activity based on an 11-point scale (0, 0.5, 1–9; 0 being the lowest-intensity activity and 9 being the highest). The intensity of activity is determined by measuring the magnitude and frequency of acceleration every 4 s. Data from participants who had worn the accelerometer for at least 10 h (600 min) a day for at least four weekdays and one weekend day were considered valid. The main physical activity variable used in this study was the duration of moderate to vigorous physical activity (MVPA). All minutes of recording with an activity level ≥4 were classified as MVPA. The activity level threshold of 4 was derived from a calibration study and corresponded to approximately three metabolic equivalents^41^. To establish whether physical activity had an effect on clock gene expression, we divided the participants into two groups namely, an active group and an inactive group. The active group included participants who performed MVPA for 150 min or more per week. This cut-off was chosen because this amount of physical activity is the minimum recommended by current physical activity health guidelines. The inactive group included participants who performed MVPA for less than 150 min per week.

### Hair follicle cells collection and laboratory assays

Following the physical activity measurements, hair follicle cells were collected from all participants. The participants were asked to refrain from consuming excess alcohol, and to maintain normal eating and sleeping habits during the sampling day. Hairs were collected over a 24 h period at 4 h intervals by firmly holding and pulling the facial hair root, and then quickly soaking the hair in dissolution buffer (RNeasy Micro Kit; QIAGEN, Hilden, Germany). More than 10 hairs were collected at each sampling point. All samples were stored at −80 °C until RNA purification. The RNeasy Micro Kit was used with frozen cytolysis solution to purify total RNA. Total RNA was reverse-transcribed using an Advanced cDNA Synthesis Kit for RT-qPCR (Bio-Rad Laboratories, Inc., CA, USA), and real-time PCR was performed using a TaqMan MGB probe (Applied Biosystems, CA, USA), and 1/20 volume of the reverse-transcription product. Data were obtained using the 7500 fast (Applied Biosystems, CA, USA) and expression was normalized to 18 S rRNA. The sequences of the primers and probes used are shown in Table 3. These primers and probe are consistent with those used in previous study^21^. To evaluate the circadian rhythm of clock gene expression and physical activity pattern, we used the cosinor method. The peak phase, amplitude, and rhythmicity of normalized data were determined using the single cosinor method program (Acro.exe version 3.5)^45^.

### Statistical analysis

Data was analyzed using Predictive Analytics Software (PASW) version 23.0 for Windows (SPSS Japan Inc., Tokyo, Japan). The Kolmogorov-Smirnov test was used to check for normal blood marker distribution. The distribution of these markers did not differ significantly from the normal. Using a one-way repeated measures ANOVA, we analyzed clock gene expression and physical activity patterns over a 24 h period. When significant main effects of time (24 h variation) were detected, we evaluated the amplitude and acrophase using the aforementioned cosinor method. Student’s t-test was used to compare differences between the active and inactive groups. Partial correlation was used to examine the relationship between step count, the amount of physical activity, 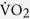 peak and expression of clock genes. Statistical significance was accepted at the 5% level. Results are presented as means ± standard error (SE).

## Additional Information

**How to cite this article**: Takahashi, M. *et al*. Positive association between physical activity and *PER3* expression in older adults. *Sci. Rep.* **7**, 39771; doi: 10.1038/srep39771 (2017).

**Publisher's note:** Springer Nature remains neutral with regard to jurisdictional claims in published maps and institutional affiliations.

## Supplementary Material

Supplemental Information

## Figures and Tables

**Table 1:** Primer sequences for real-time RT-PCR analysis and TaqMan MGB probes.

Gene	Forward	Reverse	Probe
*18S-rRNA*	CGCCGCTAGAGGTGAAATTC	CGAACCTCCGACTTTCGTTCT	CCGGCGCAAGACGGACCAGA
*PER3*	CTACCTGCACCCTGAAGATCGTTCTC	CTGGAATCCAGTATGATGTAGTCTCCGTTT	CTCTGATGGTTGCCATAC
*NR1D1*	GCTCAGTGCCATGTTCGACTTC	AAGTCTCCAAGGGCCGGTTC	AAGCTCAACTCCCTGGC
*NR1D2*	TCCAGTACAAGAAGTGCCTGAAGAATGAAA	CACGCTTAGGAATACGACCAAACCGA	ATGTCAGCAATGTCG

**Table 2:** Amplitude and acrophase of clock gene expression rhythms.

	Total (n = 20)	
	Amplitude	Acrophase
*PER3*	1.06 ± 0.08	6.84 ± 0.47
*NR1D1*	0.70 ± 0.08	4.20 ± 0.80
*NR1D2*	0.76 ± 0.07	4.76 ± 0.55

**Table 3:** Physical characteristics of the participants.

	Total (n = 20)
Age (years)	71 ± 1
Body mass (kg)	64 ± 2
Body mass index	23 ± 1
Time of breakefast (24 h)	8 ± 1
Time of lunch (24 h)	12 ± 1
Time of dinner (24 h)	19 ± 1
MEQ score	52 ± 1
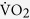 peak (ml/min)	25 ± 1
Step count (step/day)	8389 ± 802
MVPA (min/week)	170 ± 26

Data are means ± SE.
