# Supplementary material for: Positive association between physical activity and PER3 expression in older adults
Source: Sci Rep. 2017 Jan 3;7:39771. doi: 10.1038/srep39771 (PMC5206642; doi:10.1038/srep39771)
Supplement: Supplemental Information [file srep39771-s1.pdf]

Positive association between physical activity and *PER3* expression in older adults

Masaki Takahashi, Atsushi Haraguchi, Yu Tahara, Natsumi Aoki, Mayuko Fukazawa, Kumpei Tanisawa, Tomoko Ito, Takashi Nakaoka, Mitsuru Higuchi , Shigenobu Shibata

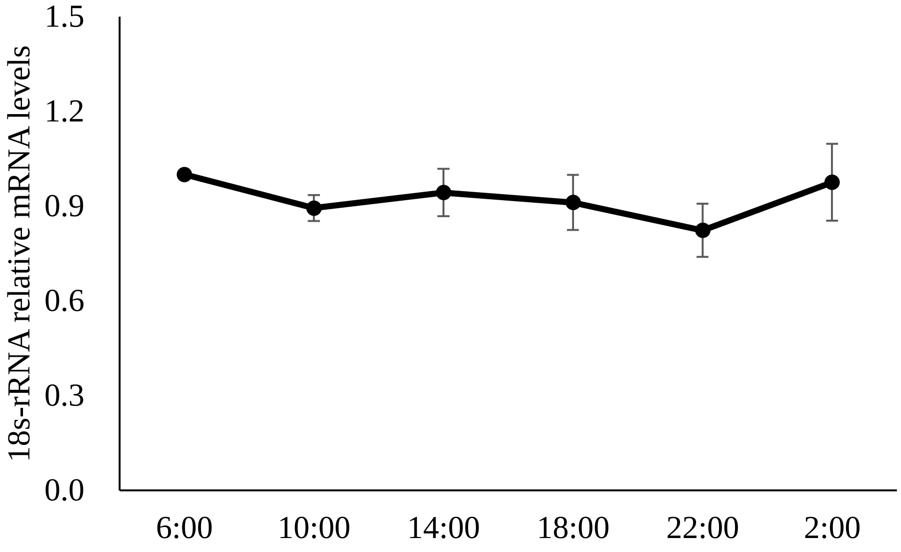

Figure S1. The diurnal 18s-rRNA expression in hair follicle cells (n = 20). Data are means ± SE.

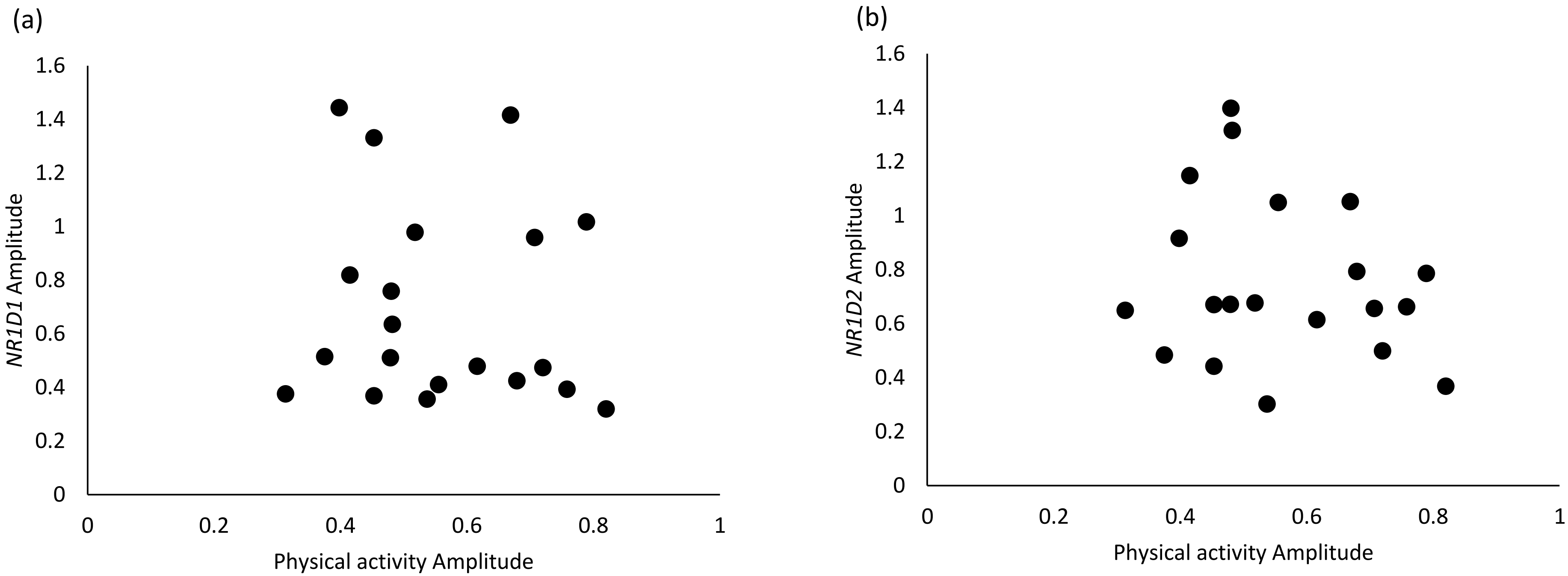

Figure S2. The relationship across all participants between amplitude of physical activity and expression of *NR1D1* (a) and *NR1D2* (b) (n = 20).

Table S1

Amplitude and acrophase of clock genes expression and physical activity in each participants

	PER3			*NR1D1*			*NR1D2*			Physical activity		
ID	Amplitude	Acrophase	Goodness of fit	Amplitude	Acrophase	Goodness of fit	Amplitude	Acrophase	Goodness of fit	Amplitude	Acrophase	Goodness of fit
1	0.910	8.8	0.083	0.960	11.6	0.094	0.657	8.0	0.316	0.707	14	0.067
2	0.874	9.2	0.066	1.332	6.0	0.106	0.443	6.8	0.020	0.453	18	0.007
3	1.475	6.8	0.100	0.759	2.0	0.309	1.398	6.0	0.135	0.48	16	0.028
4	0.987	6.0	0.113	1.416	2.0	0.225	1.052	6.0	0.107	0.669	14.8	0.016
5	0.696	6.4	0.020	0.369	2.0	0.247	0.671	2.0	0.187	0.453	16.4	0.038
6	0.914	9.2	0.095	0.320	11.2	0.349	0.368	8.0	0.506	0.82	14.4	0.072
7	1.019	2.0	0.187	1.444	1.6	0.262	0.916	2.0	0.096	0.398	15.6	0.148
8	1.588	6.8	0.075	0.411	6.0	0.189	1.049	6.0	0.022	0.555	16.4	0.033
9	1.524	2.0	0.103	0.636	1.2	0.408	1.316	1.6	0.063	0.482	15.6	0.026
10	1.025	7.2	0.063	0.820	2.0	0.062	1.149	2.0	0.225	0.415	9.6	0.195
11	1.524	6.8	0.070	0.425	2.0	0.095	0.794	6.0	0.035	0.679	16.8	0.01
12	0.703	6.0	0.129	0.511	0.4	0.239	0.672	2.0	0.294	0.479	14.8	0.073
13	0.630	6.4	0.023	0.376	7.2	0.262	0.650	6.4	0.060	0.313	15.2	0.019
14	1.145	6.0	0.051	0.479	0.0	0.053	0.615	6.0	0.124	0.616	13.6	0.058
15	0.631	6.0	0.052	0.474	2.0	0.067	0.499	2.0	0.071	0.72	14.8	0.031
16	1.753	9.2	0.150	1.018	8.4	0.207	0.787	2.0	0.148	0.789	16.4	0.003
17	1.252	8.0	0.054	0.393	6.4	0.189	0.663	8.4	0.033	0.758	13.2	0.055
18	1.194	10.0	0.157	0.979	8.0	0.043	0.677	2.0	0.205	0.518	17.6	0.017
19	0.593	6.4	0.161	0.515	2.0	0.052	0.484	6.0	0.069	0.375	16	0.037
20	0.684	7.6	0.030	0.357	2.0	0.055	0.302	6.0	0.038	0.537	14.8	0.065

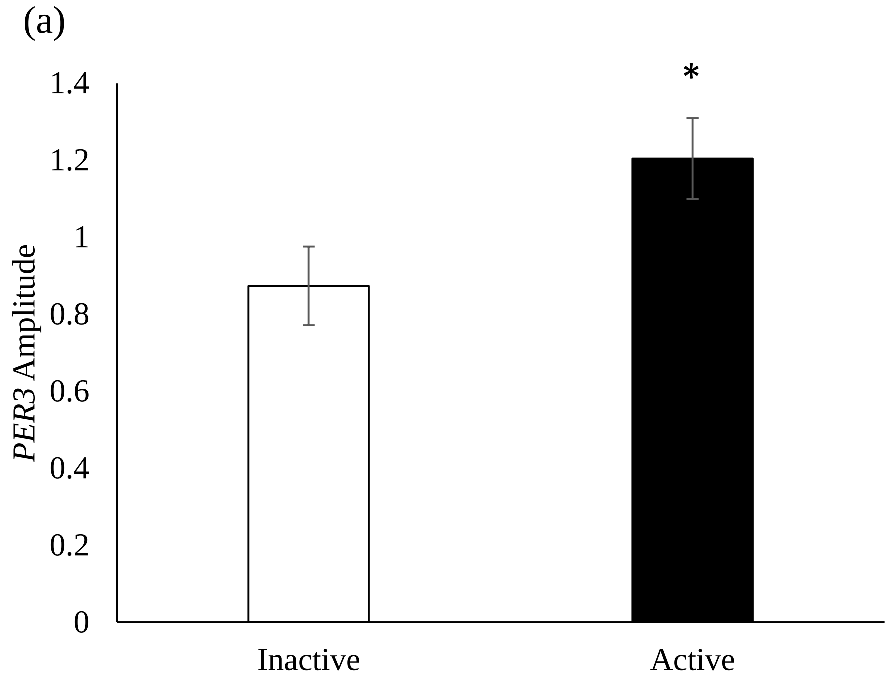

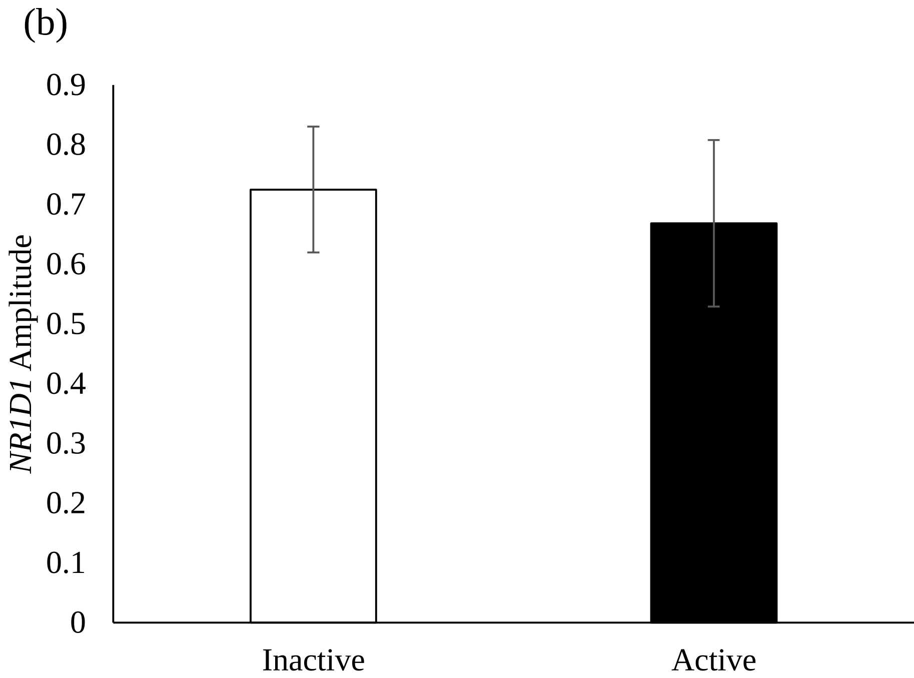

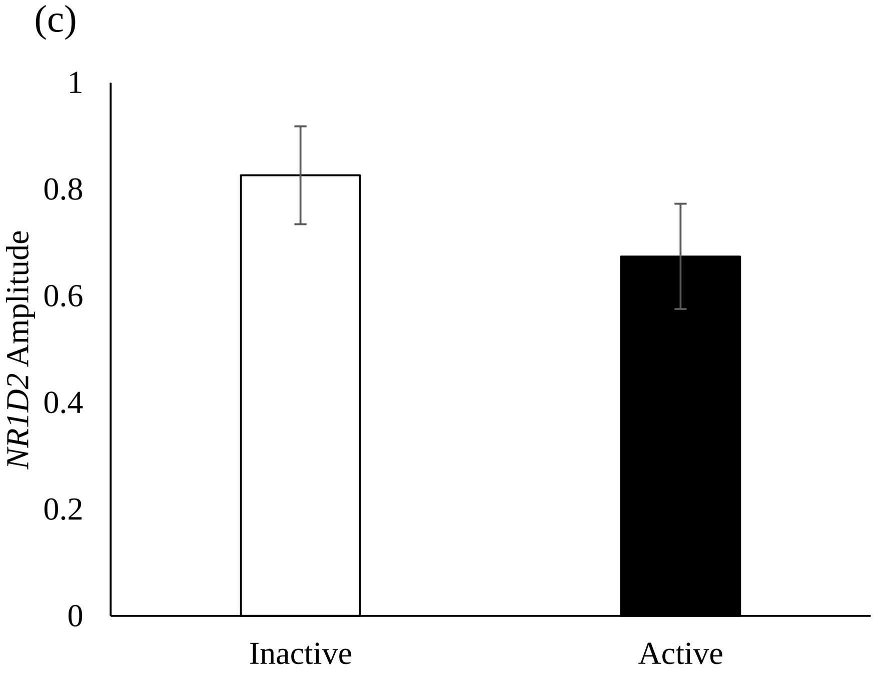

Figure S3. The amplitude of *PER3* (a), *NR1D1* (b), *NR1D2* (c) expression in the active (n = 11), and inactive (n = 9) groups. Data are means ± SE.* Significantly different from the value between active and inactive groups (unpaired Student's *t*-tests, P < 0.05).

Table S2

The relationships between physical characteristics of the participants and amplitude of clock genes expression (n = 20)

	PER3		*NR1D1*		*NR1D2*		Physical activity	
	Amplitude		Amplitude		Amplitude		Amplitude	
	r	*P*	*r*	*P*	*r*	*P*	*r*	*P*
Age（years）	-0.306	0.190	-0.129	0.587	-0.109	0.648	-0.417	0.067
Body mass（kg）	-0.092	0.701	-0.091	0.703	-0.429	0.059	0.340	0.143
Body mass index	-0.293	0.209	-0.127	0.595	-0.321	0.168	0.158	0.506
Time of breakefast (24h)	0.236	0.347	0.425	0.079	0.004	0.989	-0.124	0.623
Time of lunch (24h)	0.095	0.699	0.069	0.780	0.013	0.957	-0.009	0.971
Time of dinner (24h)	-0.401	0.080	0.073	0.761	-0.201	0.395	-0.175	0.460
MEQ score	-0.213	0.367	-0.110	0.645	-0.002	0.993	0.098	0.681
MVPA (min/week)	0.582	0.007**	0.013	0.955	0.105	0.658	0.760	0.020*
Step count (step/day)	0.383	0.096	-0.012	0.960	0.080	0.738	0.868	0.001**
VO_2 peak (ml/min)	0.481	0.032*	-0.248	0.293	0.293	0.209	0.150	0.527

Data are the Pearson's correlation coefficients.
* Significant difference (P < 0.05) , ** Significant difference (P < 0.01)

Table S3

The relationships between physical characteristics of the participants and acrophase of clock genes expression (n = 20)

	PER3		*NR1D1*		*NR1D2*		Physical activity	
	Acrophase		Acrophase		Acrophase		Acrophase	
	r	*P*	*r*	*P*	*r*	*P*	*r*	*P*
Age（years）	-0.053	0.823	-0.212	0.371	0.052	0.826	-0.226	0.337
Body mass（kg）	0.470	0.037*	0.427	0.060	-0.113	0.635	-0.020	0.933
Body mass index	0.290	0.215	0.180	0.448	-0.220	0.351	-0.216	0.260
Time of breakefast (24h)	0.288	0.247	0.127	0.615	-0.334	0.175	0.311	0.209
Time of lunch (24h)	0.276	0.253	0.051	0.836	-0.407	0.084	0.024	0.924
Time of dinner (24h)	0.049	0.837	0.181	0.445	-0.191	0.420	0.170	0.474
MEQ score	-0.204	0.387	-0.123	0.605	0.106	0.656	-0.197	0.406
MVPA (min/week)	0.458	0.042*	0.413	0.071	0.139	0.139	0.058	0.807
Step count (step/day)	0.416	0.068	0.378	0.101	0.257	0.275	-0.151	0.524
VO_2 peak (ml/min)	0.095	0.690	-0.067	0.778	0.033	0.889	0.212	0.370

Data are the Pearson's correlation coefficients.
* Significant difference (P < 0.05)
